# A RNA-Seq Analysis to Describe the Boar Sperm Transcriptome and Its Seasonal Changes

**DOI:** 10.3389/fgene.2019.00299

**Published:** 2019-04-16

**Authors:** Marta Gòdia, Molly Estill, Anna Castelló, Sam Balasch, Joan E. Rodríguez-Gil, Stephen A. Krawetz, Armand Sánchez, Alex Clop

**Affiliations:** ^1^Animal Genomics Group, Centre for Research in Agricultural Genomics (CRAG) CSIC-IRTA-UAB-UB, Campus UAB, Catalonia, Spain; ^2^Department of Obstetrics and Gynecology, Wayne State University, Detroit, MI, United States; ^3^C.S. Mott Center for Human Growth and Development, Wayne State University, Detroit, MI, United States; ^4^Unit of Animal Science, Department of Animal and Food Science, Autonomous University of Barcelona, Barcelona, Spain; ^5^Grup Gepork S.A., Barcelona, Spain; ^6^Unit of Animal Reproduction, Department of Animal Medicine and Surgery, Autonomous University of Barcelona, Barcelona, Spain; ^7^Center for Molecular Medicine and Genetics, Wayne State University, Detroit, MI, United States; ^8^Consejo Superior de Investigaciones Científicas (CSIC), Barcelona, Spain

**Keywords:** sperm, sperm RNA element, RNA-seq, sperm seasonality, transcript integrity, differential gene expression

## Abstract

Understanding the molecular basis of cell function and ultimate phenotypes is crucial for the development of biological markers. With this aim, several RNA-seq studies have been devoted to the characterization of the transcriptome of ejaculated spermatozoa in relation to sperm quality and fertility. Semen quality follows a seasonal pattern and decays in the summer months in several animal species. The aim of this study was to deeply profile the transcriptome of the boar sperm and to evaluate its seasonal changes. We sequenced the total and the short fractions of the sperm RNA from 10 Pietrain boars, 5 collected in summer and 5 five sampled in winter, and identified a complex and rich transcriptome with 4,436 coding genes of moderate to high abundance. Transcript fragmentation was high but less obvious in genes related to spermatogenesis, chromatin compaction and fertility. Short non-coding RNAs mostly included piwi-interacting RNAs, transfer RNAs and microRNAs. We also compared the transcriptome of the summer and the winter ejaculates and identified 34 coding genes and 7 microRNAs with a significantly distinct distribution. These genes were mostly related to oxidative stress, DNA damage and autophagy. This is the deepest characterization of the boar sperm transcriptome and the first study linking the transcriptome and the seasonal variability of semen quality in animals. The annotation described here can be used as a reference for the identification of markers of sperm quality in pigs.

## Introduction

### Semen Quality Is Highly Relevant for the Sustainability of Modern Pig Breeding

Swine, together with poultry, are the most important sources of meat for human consumption (in kg) worldwide (OECD, 2018). Moreover, the global demand for animal protein is growing quickly. Thus, improving the efficiency of pork production is of paramount importance for the sustainability of the sector. Pig production relies on the genetic merit of boars kept in artificial insemination centers and the quality of their sperm to disseminate their genetic material. Hence, there is an increasing demand for molecular markers that afford early prediction of semen quality and fertility in young boars.

### The Sperm Cell Contains a Complex and Functionally Relevant Transcriptome

For decades, the ejaculated mature sperm was considered a dormant cell that only carried the paternal genome to the egg. Nonetheless, in the recent years the biological complexity of sperm has become more evident, with the discovery of a rich sperm RNA population with functional roles in spermatogenesis, fertilization, early embryo development and transgenerational epigenetic transmission (Gòdia et al., 2018b). Mature sperm RNAs have been studied by NGS in several mammalian species including human (Sendler et al., 2013), horse (Das et al., 2013), mouse (Johnson et al., 2015), and cattle (Selvaraju et al., 2017). These studies have shown a sperm-specific transcriptome with a large population of transcripts most of which are present at low levels and are also highly fragmented. The sncRNA population of sperm has also been interrogated in several mammals (Krawetz et al., 2011; Das et al., 2013; Capra et al., 2017), and is composed of a large and complex repertoire of microRNAs (miRNAs), piRNAs, and tRNAs, among other RNA classes. The abundance of these transcripts has been proposed as a valuable source of bio-markers for semen quality in animal breeding and bio-medicine (Jodar et al., 2015; Salas-Huetos et al., 2015; Capra et al., 2017).

### The Boar Sperm Transcriptome

The boar sperm transcriptome has been interrogated in several studies, most employing qPCR analysis of target genes. Although qPCR is a useful tool that provides very valuable information, these studies typically assume transcript integrity and target one or two exons of only candidate genes. RNA-seq overcomes these two limitations. The first genome wide evaluation of the boar spermatozoa transcriptome was completed in 2009 by sequencing the 5′-ends of a Expressed Sequence Tag library using Sanger technology (Yang et al., 2009), which led to the identification of 514 unique sequences many of which corresponded to unknown genes. High-throughput RNA-seq was more recently applied to compare two differentially fed boars (Bruggmann et al., 2013) and to explore the short RNA component of the boar sperm (Luo et al., 2015; Pantano et al., 2015; Chen et al., 2017a; Chen et al., 2017b). These studies aimed to compare the sncRNAs at different stages of spermatogenesis or between the different components of the ejaculate, and concluded that a large proportion of these short RNAs are sperm-specific. Despite these previous studies, an in-depth analysis of the boar sperm transcriptome is still missing.

### Sperm Quality Has a Seasonal Component

Sperm quality can be influenced by multi-factorial genetics (Marques et al., 2017) and environmental factors such as stress and seasonality (Wettemann et al., 1976). In pigs, a clear drop on semen quality and male fertility has been observed in the warm summer months, possibly due to heat stress (Trudeau and Sanford, 1986; Zasiadczyk et al., 2015). This seasonal effect has been linked to altered levels of some transcripts (Yang et al., 2010).

The first step toward the efficient identification of RNA markers of sperm quality requires obtaining a profound picture of the boar sperm transcriptome. Our group has recently optimized a pipeline to extract RNA from swine mature spermatozoa and obtain a high quality and complete transcriptome profile (Gòdia et al., 2018a). In this study, we have profiled the sperm transcriptome from 10 boars, including both coding and non-coding RNAs and we have evaluated the relationship between transcript abundance and the season of collection (summer versus winter) in the northern temperate climate zone.

## Materials and Methods

### Sample Collection

Specialized professionals obtained 10 fresh ejaculates each from a different Pietrain boar from a commercial stud, with ages ranging from 9 to 28 months of age. The ejaculates were collected between July 2015 and January 2017 as previously described (Gòdia et al., 2018a). Five ejaculates were collected between December and February (winter ejaculates), and the other 5 were obtained between May and July (summer ejaculates). Fresh sperm ejaculates were obtained by the hand glove method. Spermatozoa were directly purified from the ejaculate by density gradient centrifugation (Gòdia et al., 2018a).

### RNA Extraction, qPCR Validation, Library Prep and Sequencing

RNA extraction was performed as described in Gòdia et al. (2018a). The purity of the extracted RNA, defined as RNA originating exclusively from sperm cells and devoid of DNA, was determined with three qPCR assays assessing the abundance of the sperm specific *PRM1* transcript, the somatic-cell specific *PTPRC* RNA and the presence of genomic DNA (gDNA) as previously described by our group (Gòdia et al., 2018a). RNA was then quantified with Qubit^TM^ RNA HS Assay kit (Invitrogen; Carlsbad, CA, United States) and its integrity validated with Bioanalyzer Agilent RNA 6000 Pico kit (Agilent Technologies; Santa Clara, CA, United States). Total RNA was subjected to ribosomal RNA depletion with the Ribo-Zero Gold rRNA Removal Kit (Illumina) and RNA-seq libraries were constructed with the SMARTer Low Input Library prep kit v2 (Clontech) and sequenced to generate 75 bp paired-end reads in an Illumina’s HiSeq2500 sequencing system. Short RNA-seq libraries were prepared from the same RNA aliquots (prior to rRNA depletion) with the NEBNext Small RNA (New England Biolabs) and sequenced in an Illumina Hiseq2000 to produce 50 bp single reads.

### Total RNA-Seq Mapping and Analysis of the Sperm RNA Elements

The quality of the paired-end reads were evaluated with FastQC v.0.11.1^1^, and filtered to remove low quality reads and adaptors with Trimmomatic v.0.36 (Bolger et al., 2014). Filtered reads were then mapped to the *Sus scrofa* genome (Sscrofa11.1) with HISAT2 v.2.1.0 (Kim et al., 2015) with default parameters except “–max seeds 30” and “-k 2”. Duplicate mapped reads were removed using Picard Tools^2^ MarkDuplicates. The uniquely mapped reads were used for the detection and quantification of SREs. SREs are short-size sequences characterized by a number of RNA-seq reads clustering to a given genomic location (Jodar et al., 2015; Estill et al., 2019; Gòdia et al., 2018b). This approach enables an accurate exon-quantification (or short-size sequence quantification) instead of a whole transcript mean, which makes it useful for tissues with highly fragmented RNA such as sperm. After mapping, SREs are classified as exonic (mapping to annotated exons), intronic, upstream/downstream 10 kb (if located 10 kb upstream or downstream of annotated genes) and orphan (mapping elsewhere in the genome) (Gòdia et al., 2018b). This classification was done using the pig Ensembl genome annotation (v.91) extracted with the R package “BiomaRt” (Durinck et al., 2009). Porcine orphan SREs coordinates were converted to human (hg38) coordinates and from human to bovine (bosTau8) using the UCSC liftover tool (Kuhn et al., 2013).

All the Gene Ontology enrichment analyses described throughout the article were performed with Cytoscape v.2.3.0 plugin ClueGO v.2.3.5 (Bindea et al., 2009) using Cytoscape’s porcine dataset and the default settings. Only the significant corrected *p*-values with Bonferroni were considered.

The CV of the RNA abundance across samples was used to classify the transcripts as highly unstable (CV > 0.75), moderately stable (CV between 0.25 and 0.75) and highly stable (CV < 0.25). To carry the GO analysis we used only these genes for which all their SREs fitted within the same stability class (stable, moderately stable or unstable), to ensure that genes were robustly assigned to a specific category.

### *De novo* Transcriptome Analysis

Reads unmapped to the Sscrofa11.1 genome were screened against the porcine Transposable Elements from the Repbase database (Bao et al., 2015) using HISAT2 v.2.1.0 (Kim et al., 2015). The remaining unmatched reads were searched against bacterial and viral genomes using Kraken v.0.10.5 (Wood and Salzberg, 2014) and removed. The remaining reads were subjected to *de novo* assembly with Trinity v.2.1.0 (Grabherr et al., 2011) using default parameters and databases. The assembled contigs were quantified with RSEM and only those with identity score > 85%, abundance levels > 50 FPKM and detected in 5 samples or more were kept.

### Repetitive Elements and Long Non-coding RNAs

The proportion of reads in RE was calculated with Bedtools (Quinlan and Hall, 2010) multicov using the RepeatMasker database (Bao et al., 2015). Read counts were normalized for RE length and sequencing depth. The same approach was used for lncRNAs. Only the lncRNAs annotated in Ensembl v.91 were used. The coding genes mapping less than 20 kb apart from the lncRNAs were considered as potential *cis*-regulated lncRNA targets.

### Transcript Integrity

RNA transcript integrity (TIN) was calculated with RseQC v.2.6.4 (Wang et al., 2012) using the Ensembl v.91 pig annotation. TIN indicates the proportion of a gene that is covered by reads. As an example, TIN = 100 indicates a fully covered transcript. Transcript abundance was calculated using expression.py from the same software. Transcript length was calculated based on CDS length, extracted with the R package “BiomaRt” (Durinck et al., 2009).

### Analysis of the Short Non-coding RNAs

Trimming of adaptors and low quality bases were performed with Cutadapt v1.0 (Martin, 2011) and evaluated with FastQC v.0.11.1^1^. The mapping of sncRNAs was performed with the sRNAtoolbox v.6.17 (Rueda et al., 2015) with default settings and giving as library datasets: tRNA database (Chan and Lowe, 2016), miRBase (Kozomara and Griffiths-Jones, 2011) release 21, piRNA database (Rosenkranz, 2016) and Mt tRNA, Mt rRNA, snRNA, snoRNA, lincRNA, CDS, and ncRNAs from Ensembl v.91. Multi-adjusted read counts were then normalized by sequencing depth. We only considered the miRNAs that were detected in all the samples processed. To determine if piRNAs were located in REs, the overlap between REs and the piRNA clusters that were shared in at least 3 samples was checked with Bedtools (Quinlan and Hall, 2010) multicov using the RepeatMasker database (Bao et al., 2015). The short RNA-seq reads that did not align to any of the datasets provided were used for the *de novo* piRNA annotation using ProTRAC v.2.4.0 (Rosenkranz and Zischler, 2012) and forcing a piRNA length between 26 and 33 bp and a default minimum cluster length of 5 kb. We then kept only these putative novel clusters that were shared in at least 3 of the sperm samples.

### Analysis of the Seasonal Variation of the Boar Sperm Transcriptome

We studied the potential seasonal effect of the sperm transcriptome by comparing the summer (*N* = 5) and the winter (*N* = 5) ejaculates. Total RNA-seq analysis was performed for the transcripts annotated in the pig genome. We quantified RNA abundance with the software StringTie v.1.3.4 (Pertea et al., 2015). Transcript counts were then used for the differential analysis using the R package DESeq2 (Love et al., 2014) correcting for sequencing run batch. Similarly, the identification of differential miRNAs was also carried with DESeq2 (Love et al., 2014). We only considered the differentially abundant transcripts and miRNAs with adjusted FDR values < 0.05 and FC > 1.5.

## Results and Discussion

### Total RNA-Seq Analysis: Characterization of Sperm RNA Elements

**FIGURE 1:**
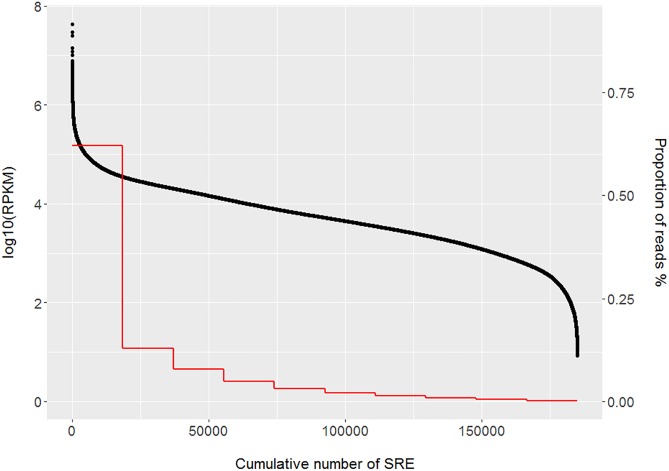
Cumulative abundance of the porcine SREs. The black dots indicate the log10 of the RNA abundance of each SRE. SREs are sorted in a decreasing order by their RNA abundance in the *X*-axis. The red line represents the total number of SREs for each abundance decile group. The first decile of the most abundant SREs accounted for 65% of the total read abundance. RPKM, reads per kilobase per million mapped reads; SRE, sperm RNA element.

RNA extraction yielded an average of 2.1 fg per cell (Supplementary File S1). These RNAs were devoid of intact ribosomal 18S and 28S RNA with RIN values below 2.5 and were free of gDNA and RNA from somatic cell origin (Gòdia et al., 2018a). On average, the total RNA-seq libraries yielded 23.6 M paired-end reads (Supplementary File S1). A total of 81.3% of the reads that passed the quality control filter mapped unambiguously to the pig genome (Supplementary File S1). After duplicate removal, a mean of 5.6 M reads per sample were obtained, resulting in a percentage of unique reads similar to recent data on human sperm (unpublished results). These reads were used for further analysis and yielded 185,037 SREs (Estill et al., 2019). Most SREs were present at low abundances but the 10% most abundant (top decile) SREs accounted for 65% of the read count with RNA levels ranging between 83 and 378,512 RPKM (Figure 1). Most of these top decile SREs were exonic (Supplementary File S2). Notably, the majority (65%) of the intronic and upstream/downstream 10 kb SREs mapped in or near genes that also harbored exonic SREs. The exonic, intronic and the upstream/downstream 10 kb top decile SREs mapped in or near 4,436 annotated genes, which were thus considered to be abundant in the boar sperm transcriptome (Supplementary File S2).

The number of annotated genes with allocated SREs in our study in swine is very similar to what has been found in the human sperm (4,765) using the same SRE bioinformatics approach (Estill et al., 2019). As expected, the number of genes identified in sperm is notably low when compared to other porcine tissues. The number of expressed genes reported in porcine muscle (Chen et al., 2011), liver (Chen et al., 2011), fat (Chen et al., 2011; Corominas et al., 2013), pituitary glands (Shan et al., 2014), hypothalamus (Pérez-Montarelo et al., 2014), duodenum (Mach et al., 2014), ilium (Mach et al., 2014) and pre-pubescent male gonads (Esteve-Codina et al., 2011), ranges between 12,816 and 18,878 genes. Among these, the immature male gonads displayed the lower number of reported expressed genes. Although these values can be only taken as a guide because each study carried their own experimental pipeline, they are indicative that the boar sperm contains a way less rich and complex transcriptome when compared to other tissues.

The top decile SREs also included 2,667 orphan SREs (SREs located more than 10 kb apart from the closest annotated gene) (Supplementary File S2). However, nearly 30% of the orphan SREs mapped within 30 kb from the closest gene, which indicates that, as the novel upstream/downstream 10 kb SREs, they may represent unannotated exons of these genes. In summary, only 10% of the top decile SREs were not linked to annotated genes. A recent study carried by Pertea et al. (2018) analyzed RNA-seq data from 9,795 human experiments from the GTEx project and concluded that the human genome annotation incorporates most of the *Homo sapiens* genes but still lacks a large proportion of the splice isoforms. While this study increased the list of coding genes by only 5%, the catalog of splice isoforms grew by 30%. Our data is in line with these recent results and does not only indicate that the novel annotation of the pig genome annotation incorporates most of the genes found in sperm but also reveals that there is still a large amount of splice isoforms to be discovered in this species. Since it is well known that the spermatozoon harbors a very specific transcriptome, a large proportion of these unannotated isoforms are likely to be sperm-specific (Sendler et al., 2013; Ma et al., 2014).

In order to dig further into the porcine sperm transcriptome, we investigated whether the porcine orphan SREs could correspond to genes not annotated in the pig but annotated in the human or cattle genomes. To this end, the location in the pig genome of the 2,667 orphan SREs were liftover onto the human and bovine genome coordinates. This resulted in 1,505 (56.4% of the 2,667 orphan SREs) human and 1,313 (49.2%) bovine syntenic regions. Forty five of the genes annotated within these regions were detected in both human and cattle (Supplementary File S3), including *CDYL*, a gene implicated in spermatid development and *ANXA3*, which protein levels in sperm have been found altered in men with poor semen compared to men with good sperm quality (Netherton et al., 2018). Ontology analysis of the 4,436 most abundant genes together with the 45 orphan SRE orthologs showed an enrichment of the cellular protein metabolic process (*q*-value: 2.7 × 10^−12^), macromolecular complex subunit organization (*q*-value: 2.1 × 10^−9^), sexual reproduction (*q*-value: 6.5 × 10^−8^), spermatogenesis (*q*-value: 1.2 × 10^−6^) and male gamete generation (*q*-value: 1.4 × 10^−6^), among others (Supplementary File S4). The transcripts detected in our study are concordant with previous results in human (Jodar et al., 2016) and bovine (Selvaraju et al., 2017) sperm and included genes related to fertilization (e.g., *HSPA1L* and *PRSS37*) or spermatogenesis (*ODF2* and *SPATA18*).

The top 30 most abundant annotated protein coding SREs mapped to 27 genes (Table 1), 12 from mitochondrial origin (e.g., *COX1*, *COX2*, *ATP8*, *ATP6*, and *COX3*), and 15 encoded in the nuclear genome (e.g., *PRM1*, *OAZ3*, *HSPB9*, and *NDUFS4*). The abundance of mitochondrial genes reflects the high number of mitochondria typically contained in a spermatozoa cell to provide critical functions for the cell’s fertilizing ability including energy supply, regulation of molecular mechanisms involved in the development of the capacitation process, production of reactive oxygen species and calcium homeostasis (Rodriguez-Gil and Bonet, 2016). The 15 nuclear genes included members related to spermatogenesis, chromatin compaction and embryo development (Sendler et al., 2013; Selvaraju et al., 2017).

**Table 1:** List of the 30 most abundant SREs in the porcine sperm.

Ensembl ID	Gene ID	SRE genomic coordinates	SRE type	Mean abundance	Abundance SD
ENSSSCG00000018075	*COX1*	MT:6511–8055	EXON	42244	14055
ENSSSCG00000018078	*COX2*	MT:8203–8890	EXON	25411	11931
ENSSSCG00000018080	*ATP8*,				
ENSSSCG00000018081	*ATP6*,	MT:8959–10583	EXON	18282	10076
ENSSSCG00000018082	*COX3*				
ENSSSCG00000021337	*PRM1*	3:31861071–31861233	EXON	14509	2711
ENSSSCG00000018094	*CYTB*	MT:15342–16481	EXON	13414	6153
ENSSSCG00000018091	*ND5*	MT:12935–14755	EXON	12285	7527
ENSSSCG00000027091	*OAZ3*	4:97442381–97442556	EXON	10492	2592
ENSSSCG00000027091	*OAZ3*	4:97441308–97441393	EXON	10441	3563
ENSSSCG00000018092	*ND6*	MT:14739–15266	EXON	8983	5521
ENSSSCG00000016203	*CFAP65*	15:121057113–121057202	NOVEL_INTRONIC	8302	7705
ENSSSCG00000018086	*ND4*,	MT:11069–12736	EXON	7984	4396
ENSSSCG00000018087	*LND4*				
ENSSSCG00000006302	*GPR161*	4:82900699–82900818	EXON	7256	1350
ENSSSCG00000018069	*ND2*	MT:5087–6128	EXON	7038	4966
ENSSSCG00000027091	*OAZ3*	4:97443314–97443450	EXON	6469	1151
ENSSSCG00000006688	*ANKRD35*	4:99454337–99454374	EXON	6130	1564
ENSSSCG00000028031	*HDAC11*	13:70866593–70866635	EXON	6012	820
ENSSSCG00000005585	*DENND1A*	1:264683712–264683755	EXON	5849	1643
ENSSSCG00000006302	*GPR161*	4:82896938–82897042	EXON	5714	839
ENSSSCG00000017609	*ANKFN1*	12:32508908–32509087	NOVEL_INTRONIC	5539	3823
ENSSSCG00000006688	*ANKRD35*	4:99459430–99459495	EXON	5483	1332
ENSSSCG00000007010	*ZMAT4*	17:9836268–9836357	NOVEL_INTRONIC	5411	4921
ENSSSCG00000017770	*PROCA1*	12:44943383–44943515	EXON	5242	1245
ENSSSCG00000017413	*HSPB9*	12:20636767–20637249	EXON	5235	1007
ENSSSCG00000000018	*KIAA0930*	5:4184013–4184090	EXON	5176	1151
ENSSSCG00000018065	*ND1*	MT:3922–4876	EXON	5155	3419
ENSSSCG00000021337	*PRM1*	3:31861339–31861529	EXON	5137	988
ENSSSCG00000016893	*NDUFS4*	16:32891178–32891257	NOVEL_INTRONIC	4843	2985
ENSSSCG00000023974	*PHF21A*	2:16386945–16386977	EXON	4792	1481
ENSSSCG00000006688	*ANKRD35*	4:99450478–99450566	EXON	4760	753
ENSSSCG00000035537	*RUNX1*	13:198392909–198392938	NOVEL_INTRONIC	4759	5935

*The most abundant SREs from protein coding genes included 12 mitochondrial and 15 nuclear genes. Some genes (e.g., PRM1, OAZ3, and ANKRD35) presented more than one highly abundant SRE. SD, standard deviation; SRE, sperm RNA element. The SRE genomic coordinates are displayed in the format chromosome:start location–end location. Mean abundance and abundance SD are indicated in RPKM: reads per kilobase per million mapped reads.*

### Total RNA-Seq Analysis: Variance on the SRE Abundance

We evaluated the transcripts that contained the 10% most abundant SREs across all samples and classified them as uniform (coefficient of variation or CV < 25%) or variable (CV > 75%). This identified 481 genes for which all their SREs were uniformly represented (CV < 25%) and 276 genes where each SRE was highly variable (CV > 75%). The list of 481 genes with constant abundance was enriched for several functions including the regulation of calcium, ATP generation and spermatid development and differentiation (Supplementary File S5). On the contrary, the highly variable genes were only enriched for the gene ontology term: single fertilization (zygote formation), which includes *SPMI*, *AQN-1* and *BSP1* among others (Supplementary File S5). This transcript variability is in general tolerated because it does not have severe phenotypic consequences. However, some of these transcripts may incur in a significant impact on semen quality and/or fertility and they could thus be biomarkers of the boar’s reproductive ability. Thus, it would be worth exploring the relationship between these genes and reproductive phenotypes in a larger study.

Jodar et al. (2016) compared the transcriptome of testes, sperm and seminal fluid and classified the corresponding transcripts according to their relative abundance in these tissues. Subsequently, they used this classification to partition the transcripts that are present in sperm into testes-enriched, sperm–enriched and seminal fluid-enriched fractions. Testes-enriched transcripts are those that presented more than 40 FPKM in testes and less than 10 FPKM in sperm and seminal fluid. The same principle applied to the other two fractions.

According to this partition (Jodar et al., 2016), we identified in our porcine dataset, 728 testes, 448 seminal fluid and 381 sperm–enriched SREs. We compared the abundance variability of these three SRE categories and found no difference between the sperm-enriched and the other fractions (Tukey’s ‘Honest Significant Difference,’ *p*-values: 0.18–0.20). However, we detected a significant difference between the testes-enriched and the seminal fluid-enriched SREs (*p*-value: 3.6 × 10^−4^). The seminal fluid-enriched fraction was, in average, more variable. The difference on the abundance variability between the testes-enriched and the seminal fluid-enriched fraction might have a biological explanation. Spermatogenesis is a finely orchestrated multi-step process that occurs in the testis, which may require a stable set of transcripts in each of these steps. On the contrary, the seminal fluid-enriched transcripts are likely to have been infiltrated into sperm via seminal exosomes (Vojtech et al., 2014; Jodar et al., 2016). The exosome uptake process may be relatively prone to variability as it is influenced by the concentration of exosomes in the seminal fluid, the RNA-load within these exosomes, and the efficiency in which the exosomes are merged with and release their content into the sperm cells.

### Total RNA-Seq Analysis: Transcript Integrity

Sperm transcripts have been found to be highly fragmented in several mammalian species (Das et al., 2013; Sendler et al., 2013; Selvaraju et al., 2017; Gòdia et al., 2018a). We sought to investigate whether this fragmentation followed a programmatic pattern or perhaps was stochastic in the pig. For each annotated transcript, we calculated the abundance levels (in FPKM) and the TIN. In average, we found 31,287 protein coding transcripts with FPKM > 0 and TIN values > 0. Most transcripts (55%) were highly fragmented (TIN ≤ 25) whilst only 181 were almost intact (TIN > 75). Interestingly, the 10 samples showed similar TIN patterns across transcripts (Pearson correlation 0.72–0.93) (Supplementary File S6). The correlations between TIN and transcript length and transcript abundance were low (0.14–0.20 and 0.14–0.25, respectively) (Supplementary File S6).

We then searched for gene ontology enrichment using the 10% most abundant transcripts within each TIN group. The highly fragmented group (TIN < 25) was enriched for genes related to negative regulation of JNK cascade (*q*-value = 1.2 × 10^−3^), spindle assembly (*q*-value = 5.6 × 10^−3^), and regulation of DNA repair (*q*-value = 4.5 × 10^−3^), among others. These results are comparable to a previous study in human sperm (Sendler et al., 2013), where the most fragmented transcripts were not enriched for spermatogenesis or fertility functions. On the other hand, no significant pathways were found in the group of the top 10% most intact transcripts, possibly due to the low size of this group (18 transcripts), even though it contained genes related to spermatogenesis (*PRM1*, *OAZ3*, and *ACSBG2*), sperm movement (*PRM3* and *SMCP*) or heat stress response (*HSPB9*) (Table 2). Remarkably, the six aforementioned genes were also within the most intact transcripts in human sperm (Sendler et al., 2013), thereby indicating conservation across species and their likely basic function in supporting sperm development and/or fecundity. Altogether, this indicates that the transcript fragmentation typically found in sperm may follow a programmatic basis and possible owe to relevant functions during spermatogenesis or upon fertilization.

**Table 2:** List of the 10% most abundant intact transcripts (TIN > 75) in the boar sperm.

Ensembl Transcript ID	Gene ID	TIN mean	TIN SD
ENSSSCT00000018955	*ZNRF4*	97.60	0.56
ENSSSCT00000007842	*TMEM239*	93.54	2.54
ENSSSCT00000019381	*HSPB9*	92.62	3.48
ENSSSCT00000046661	*UBL4B*	91.93	2.24
ENSSSCT00000001702	*C6orf106*	89.91	4.00
ENSSSCT00000006503	*SPATC1*	86.40	2.61
ENSSSCT00000030220	*OAZ3*	84.91	2.74
ENSSSCT00000004015	*AZIN2*	83.92	3.15
ENSSSCT00000049885	*PRM3*	83.56	2.27
ENSSSCT00000029296	*DBIL5^∗^*	83.21	3.71
ENSSSCT00000014766	*ZNRF4*	82.36	2.23
ENSSSCT00000048242	*ACSBG2^∗^*	81.15	2.62
ENSSSCT00000007224	*SMCP*	79.47	5.33
ENSSSCT00000003898	*KIF17*	79.04	1.67
ENSSSCT00000007327	*ANKRD35*	78.97	2.91
ENSSSCT00000012714	*DNAJB8*	76.43	4.11
ENSSSCT00000000746	*TPI1*	75.99	3.63
ENSSSCT00000029974	*PRM1*	75.72	1.44

*Transcript integrity was measured as TIN, transcript integrity number; TIN mean, Average TIN. SD, standard deviation. ^∗^Gene symbol extracted from an orthologous gene species.*

### Total RNA-Seq Analysis: *De novo* Transcriptome Assembly

We sought to further exploit the RNA-seq data by performing *de novo* assembly of the reads that did not map to the porcine genome. An average of 5.1 M unmapped reads per sample were used for the analysis (Supplementary File S1) and assembled into a mean of 8,459 contigs per sample, with a median size (N50) of 259 bp (Supplementary File S7). These contigs were then contrasted by sequence homology against several protein databases and after filtering, resulted in a list of 1,060 proteins from human, cattle, mouse, pig, and other animal species with moderate to high RNA abundance (Supplementary File S8). Some of the proteins were detected in more than one species and accounted for a total of a non-redundant list of 768 unique genes (Supplementary File S9). The majority of these genes (739) were already present in the porcine annotation whilst 29 were classified as novel genes. From the annotated genes, 699 were also detected with our initial pipeline mapping the SREs to the porcine genome but 40 were only detected by this *de novo* assembly (Supplementary File S9).

The unmapped reads that found a gene that is annotated in swine in the *de novo* analysis, could have remained unmapped due to two main reasons. They could have either harbored more mismatches than the maximum allowed for the mapping algorithm, or they might have corresponded to genomic segments not assembled to the current version of the porcine genome. We re-mapped the unmapped reads employing the looser mismatch penalty scores (the default is 6) 5, 4, 3, and 2 and obtained a small improvement in the read mapping percentage (92.9, 88.5, 85.8, and 77.3% of the reads remained unmapped reads, respectively). This shallow increase in the read mappeability suggests that a large proportion of the unmapped reads might have corresponded to genomic regions that are not assembled in the current version of the swine genome.

The 40 known genes detected only by the *de novo* assembly together with the 29 potential novel genes did not cluster into any GO biological process. However, some of these genes have been associated to spermatogenesis or implicated in the sperm structure such as the sperm head or flagellum (e.g., *ACSBG2*, *HSF2BP*, *CCNYL1*, *KNL1*, and *WBP2NL*). These results are in line with the recent study carried in humans by Pertea et al. (2018) as already detailed in relation to the orphan SREs. Although the number of novel protein-coding genes represents a modest increase (29 genes), our *de novo* analysis yielded a much higher number (699) of potentially novel splice variants.

### Total RNA-Seq Analysis: Repetitive Elements

Repetitive elements (REs) are of particular interest as they comprise a high proportion of the porcine genome (approximately 40%) often related to genome instability (Bzymek and Lovett, 2001). Germline cells are very sensitive to the deleterious effects of active transposable elements. For example, the disruption of LINE1 retrotransposon silencing, the most abundant RE in the pig genome, can lead to spermatogenesis aberrations (Gòdia et al., 2018a) and embryo development arrest (Beraldi et al., 2006). Due to their relevance in spermatozoa, we annotated the RE segments that were transcribed in the pig sperm. A total of 4.6% of the mapped reads overlapped with REs, which is in line with previous data in murine sperm (Johnson et al., 2015), and accounted for 42.8 Mb of the swine genome. The most enriched RE classes included simple repeats (2.58% of the total mapped reads) which could potentially correspond to porcine nuclear matrix associated RNAs (Johnson et al., 2015). The second most abundant REs were the SINEs which accounted for 0.6% of the total read abundance. SINEs are transposable elements that can be hypo-methylated and can regulate male germ cell development, sperm packaging and embryo development (Schmid et al., 2001). In pigs, LINE1 accounts for 16.8% of the genome space and in our study, 0.19% of the mapped reads overlapped with LINE1 segments and spanned 25.5 Mb of the genome. This is nearly ten times less than in mice (1.89%) (Johnson et al., 2015) even though LINE1 is just slightly more ubiquitous in the murine genome (20%) (Waterston et al., 2002). While potentially interesting, these differences may arise due to yet unknown species-specific biological particularities or technical differences in the library preparation and/or bioinformatics methods used in both studies.

### Total RNA-Seq Analysis: Long Non-coding RNAs

Long non-coding RNAs are regulatory RNAs above 200 bp long implicated in a plethora of functions, including spermatogenesis and reproduction (Gòdia et al., 2018b). Sperm lncRNAs have been reported in human (Sendler et al., 2013), mice (Zhang et al., 2017), and cattle (Selvaraju et al., 2017). We identified 27 of the 361 lncRNA annotated in Ensembl v.91, and their RNA levels were clearly below their coding SRE counterparts (Supplementary File S10). The predicted *cis*-regulated target genes included *ZNF217*, which is a transcriptional repressor, *DYNLRB2* which encodes for a protein belonging to the dynein family of axoneme components related to sperm motility and *YIPF5*, which caused infertility in a knock-out fruit fly model (Yu et al., 2015). The annotation of lncRNAs in the swine genome remains remarkably poor and here we provide an initial catalog that is still incomplete.

### Short RNA-Seq Analysis

**FIGURE 2:**
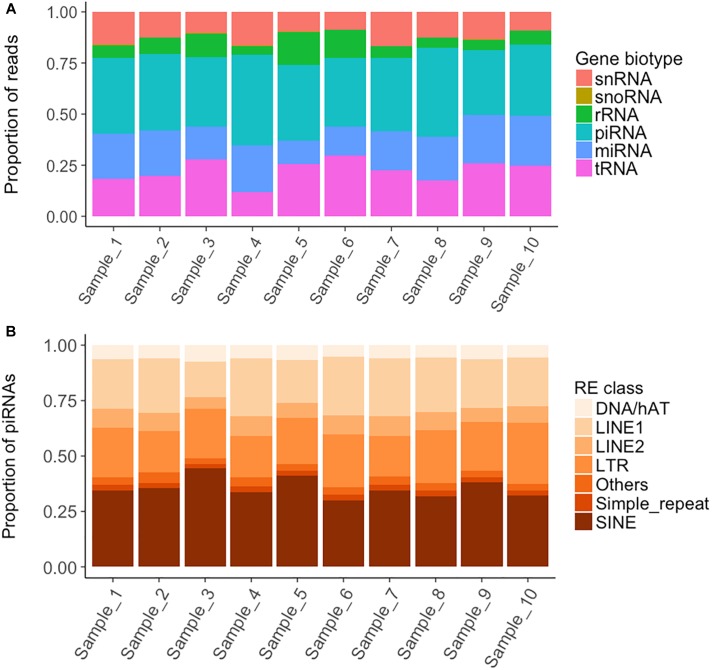
Read mapping distribution of the short non-coding RNA types and piRNA distribution within the Repetitive Element classes. **(A)** Proportion of reads mapping to each short non-coding RNA type. **(B)** Distribution within each Repetitive Element class of the piRNA cluster reads overlapping with Repetitive Elements.

On average, 6.6 M reads were obtained for each short RNA-seq library. A mean of 83% of these reads aligned to the queried porcine (*S. scrofa*) databases (Supplementary File S1). A total of 34% of the aligned reads corresponded to sncRNAs, mainly piRNAs (37% of the sncRNA fraction), tRNAs (22.6%) and miRNAs (20.2%) (Figure 2A and Supplementary File S11). The remaining aligned reads (66%) mostly belonged to mitochondrial transfer and ribosomal RNAs (51%) but also to nuclear protein coding genes (Supplementary File S11).

The functional relevance of miRNAs, piRNAs, and tRNAs in sperm biology and fertility (Krawetz et al., 2011; Sharma et al., 2016; Gòdia et al., 2018b) is well known. miRNAs are a class of sncRNAs that have been found in multiple cell types and involved in a plethora of phenotypes and diseases. They post-transcriptionally repress the translation of target messenger RNAs (mRNAs) and can be ideal biomarkers for many traits including sperm quality and fertility. We detected 105 miRNAs (annotated in the pig) that were present in all the samples, with an average abundance that ranged from 4.6 to 13,192.2 CPMs. Chen et al. (2017a) carried a RNA-seq study using one pool of 3 pig sperm samples and detected a larger number of miRNAs -140- than in our study, but the overlap was remarkable, with 75 of the 140 miRNAs found in both experiments (Supplementary File S12). The lower number of miRNAs described in our work compared to Chen et al. (2017a), is somewhat not surprising as we only considered those miRNAs that were present in the 10 samples and used thus more stringent parameters. The inter-species comparison also indicates a degree of conservation in the miRNA composition of the mammalian sperm with about 70% of the miRNAs shared in cattle (Capra et al., 2017) and human (Pantano et al., 2015) (Supplementary File S12). These results suggest a conserved functional role of these miRNAs in mammals. The most abundant miRNAs in our study, miR-34c, miR-191, miR-30d, miR-10b and let-7a, among others (Supplementary File S13), are also highly abundant in cattle (Capra et al., 2017) and in human (Krawetz et al., 2011; Pantano et al., 2015) sperm. Some of these miRNAs have been linked to the male’s reproductive ability. For example, miR-34c is crucial for spermatogenesis (Yuan et al., 2015) and has been related to bull fertility (Fagerlind et al., 2015) and miR-191, miR-30d, and miR-10b displayed altered levels in infertile human patients when compared to healthy controls (Salas-Huetos et al., 2015; Tian et al., 2017).

We then assessed the CV across the sperm samples to evaluate their abundance stability (Supplementary File S13). Interestingly, miRNAs showed large variability, 32% of them varied markedly (CV > 75%), including the highly abundant miR-34c, miR-30c-5p, miR-186, and miR-99a, with none showing low variability. As previously mentioned, exosome vesicles may also contribute in modulating the miRNA population of recipient cells. In fact, a recent study identified altered miRNA profiles in seminal plasma exosomes from azoospermic patients (Barcelo et al., 2018). We did not measure the pairwise correlation between the abundance of miRNAs and mRNAs because in their canonical function, miRNAs inhibit translation but have a small impact on the levels of the target mRNAs.

piRNAs are a class of 26-32 bp size sncRNAs that interact with Piwi proteins to contribute important functions to germline development, epigenetic regulation and the silencing of transposable elements (O’Donnell and Boeke, 2007). We queried a public database of 501 piRNA clusters identified in pig testes (Rosenkranz, 2016), and found that 300 were represented in boar sperm and covered 5.03 Mb (0.20%) of the Scrofa10.2 genome assembly (Supplementary File S13). The RNA levels ranged between 3.2 and 5,242 CPMs and the cluster length between 5,077 and 114,717 bp. piRNA clusters tend to overlap with REs, in keeping with their role in genome inactivation and transposon regulation (O’Donnell and Boeke, 2007; Krawetz et al., 2011; Pantano et al., 2015; Gòdia et al., 2018b). In our work, 25% of the piRNA clusters co-localized with REs, most of which were SINEs (Figure 2B). As piRNAs are tissue-specific and we queried a testes database (Rosenkranz, 2016), we also carried a *de novo* prediction of piRNA clusters with proTRAC using the remaining unaligned reads (average of 1.1 M reads) (Supplementary File S1). We identified 17 novel potential clusters of average abundance and length of 11.3–585 CPMs and 2,357–56,029 bp, respectively, and as a whole, they covered 159.7 kb of the Sscrofa11.1 genome. Six of the novel clusters were present in the 10 samples and are thus considered of high confidence (Supplementary File S14).

tRNAs were the second most abundant RNA class in porcine sperm, and their abundance is related to metabolic processes (Sharma et al., 2016). We identified 315 putative tRNAs from which 63% showed large abundance variability across samples (CV > 75%) (Supplementary File S13). Although the role of tRNAs in germ cells and in the offspring’s health is uncertain, independent studies have shown that tRNA levels can be altered in response to certain manipulations of the paternal diet (Sharma et al., 2016; Gòdia et al., 2018b).

### Seasonal Differences in the Boar Sperm Transcriptome

A seasonal variation on semen quality and fertility has been observed in several animal species including the pig. During the warm summer months, as the scrotum is unable to thermo-regulate, spermatogenesis is negatively affected and the number of sperm cells and their motility tend to decrease alongside with an increase on morphological abnormalities (Zasiadczyk et al., 2015; Rodriguez et al., 2017). This effect on semen quality and also fertility (Suriyasomboon et al., 2006) has been related to heat stress. The molecular mechanisms underlying this phenomenon remain unclear although links to oxidative stress and the production of reactive oxidative species (ROS), with the consequent damage on sperm membrane integrity, DNA damage, apoptosis, autophagy and reduction of mitochondrial activity have been proposed (Durairajanayagam et al., 2015; Argenti et al., 2018). In a recent study, Argenti et al. (2018) identified increased superoxide dismutase anti-oxidant activity in the sperm of boars raised in sub-tropical Brazil in the summer months probably as a molecular attempt to reduce the presence of ROS and sperm damage (Argenti et al., 2018). Moreover, dietary strategies based on supplementary Zinc (Li et al., 2017) and l-arginine (Chen et al., 2018) have been related to a reduction of oxidative stress and improvement on the epididymal function and boar sperm quality in summer.

We compared the transcriptome (mRNA transcripts and miRNA) of the sperm samples collected in the summer months (May: *N* = 1; July: *N* = 4) with those collected in winter (December: *N* = 2; January: *N* = 2; February: *N* = 1) in a temperate climate zone (latitude 42° N, 800 m above sea level) with average temperatures in December–February around 2–3°C, 12°C in May and 19°C in July, but which easily peaks to highs above 30°C during this month (data from Sant Pau de Segúries weather station according to the Meteorological Service of Catalonia). The semen quality of the summer and winter groups was not significantly different when compared with a *T*-test, although a trend was seen for sperm cell viability (*p*-value = 0.05), acrosome reaction (*p*-value = 0.09) and neck (*p*-value = 0.07) and tail (*p*-value = 0.08) morphological abnormalities. We detected 36 transcripts displaying a significant difference in abundance. Of these, two transcripts corresponded to the same gene and they were not taken into account due to concerns on the transcript allocation carried by the software. From the 34 remaining transcripts, each from a different gene, 14 were up-regulated and 20 were down-regulated in the summer group (Table 3).

**Table 3:** Messenger RNA transcripts showing differential abundances in the summer versus the winter ejaculates.

Transcript ID	Gene ID	Log2 (FC)	*p*-value	*q*-value (FDR)
ENSSSCT00000058763	*NSUN6*	−9.62	7.00E-16	4.35E-12
ENSSSCT00000056639	*ATG16L1*	−7.75	1.03E-08	2.14E-05
ENSSSCT00000059752	*EHBP1*	−7.49	7.95E-08	1.35E-04
ENSSSCT00000059921	*CENPC*	−6.55	8.56E-07	1.06E-03
ENSSSCT00000012060	*MTPAP*	−6.51	3.16E-07	4.21E-04
ENSSSCT00000056608	*SMARCA2*	−6.48	1.66E-05	1.15E-02
ENSSSCT00000066205	*CNOT3*	−6.22	1.75E-06	1.72E-03
ENSSSCT00000014560	*KIF18A*	−6.01	6.79E-05	3.62E-02
ENSSSCT00000057538	*ZNF24*	−5.88	4.01E-05	2.34E-02
ENSSSCT00000018135	*AOAH*	−5.47	2.54E-05	1.58E-02
ENSSSCT00000015909	*PSMD13*	−3.76	1.60E-05	1.15E-02
ENSSSCT00000037719	*STARD9*	−2.63	2.18E-05	1.45E-02
ENSSSCT00000039055	*CPEB3*	−2.32	4.98E-05	2.81E-02
ENSSSCT00000039293	*MED13L*	−2.13	1.62E-05	1.15E-02
ENSSSCT00000043522	*OSGIN1*	−1.75	9.49E-06	7.69E-03
ENSSSCT00000012151	*CUL2*	1.66	5.66E-05	3.10E-02
ENSSSCT00000001457		4.44	6.31E-14	2.94E-10
ENSSSCT00000049515	*ZMYND10*	4.72	1.26E-06	1.31E-03
ENSSSCT00000011652	*TRUB1*	4.93	2.33E-05	1.50E-02
ENSSSCT00000049377	*NUP58*	5.14	9.56E-05	4.95E-02
ENSSSCT00000007716	*MCM8*	5.15	1.68E-20	3.13E-16
ENSSSCT00000035098	*ERBIN*	5.31	9.92E-06	7.71E-03
ENSSSCT00000031111	*ANKRD6*	5.53	2.58E-06	2.40E-03
ENSSSCT00000038311	*MCPH1*	5.65	4.31E-06	3.83E-03
ENSSSCT00000018344	*WDR70*	5.72	1.04E-06	1.18E-03
ENSSSCT00000037667	*ASCC1*	5.78	2.80E-05	1.69E-02
ENSSSCT00000002542	*FUT8*	6.00	5.14E-06	4.36E-03
ENSSSCT00000032033	*TMEM230*	6.01	2.08E-07	2.98E-04
ENSSSCT00000050364	*PDE3B*	6.45	1.62E-07	2.52E-04
ENSSSCT00000015769	*FBXO38*	6.48	3.51E-08	6.54E-05
ENSSSCT00000043281	*ZNF280D*	6.49	1.08E-06	1.18E-03
ENSSSCT00000064492	*ZNF629*	6.73	2.55E-09	6.80E-06
ENSSSCT00000028805	*ZNF583*	7.34	6.81E-10	2.12E-06
ENSSSCT00000030081	*NMNAT1*	7.50	1.59E-11	5.94E-08
ENSSSCT00000039133	*ATG16L1*	7.76	4.16E-09	9.69E-06
ENSSSCT00000038377	*RUNDC3B*	8.96	3.72E-16	3.47E-12

*The list includes only these transcripts with q-value < 0.05 and log2 (FC) < −1.5 or >1.5. log2 (FC) > 0 indicate up-regulation in winter when compared to summer. Empty cells in the Gene ID column correspond to transcripts without gene symbol or description. FC, fold-change; FDR, false discovery rate.*

The most significant difference in gene abundance between both seasonal groups (*q*-valure = 3.13 × 10^−16^, FC = 5.15) corresponded to the minichromosome maintenance 8 homologous recombination repair factor (*MCM8*) gene (Table 3). *MCM8* is a helicase related to the initiation of eukaryotic genome replication and may be associated with the length of the reproductive lifespan and menopause. *MCM8* plays a role in gametogenesis due to its essential functions in DNA damage repair via homologous recombination of DNA double strand breaks (Lutzmann et al., 2012).

Another gene was StAR Related Lipid Transfer Domain Containing 9 (*STARD9*), which was down-regulated in the winter group, is a lipid binding gene that has been related to asthenospermia in humans (Mao et al., 2011). Moreover, the paralog *STARD6* has been linked to spermatogenesis and spermatozoa quality (Mao et al., 2011). This is in keeping with the fact that the spermatozoon is very sensitive to oxidative damage for several reasons including the high amount of the peroxidation-prone unsaturated fatty acids that are present in its plasma membrane (Aitken and De Iuliis, 2010). Another gene that was found down-regulated in the winter group is the Oxidative Stress Induced Growth Inhibitor 1 gene (*OSGIN1*). *OSGIN1* has been related to autophagy and oxidative stress and its encoded protein regulates both cell death and apoptosis in the airway epithelium (Sukkar and Harris, 2017). Its expression is induced by DNA damage, which is one of the key sperm parameters that increase in the warm summer months (Perez-Crespo et al., 2008). Since this gene has also been identified in the sperm lineage, it could respond with a similar anti-oxidative role in front heat stress in sperm.

The presence of RNA differences in ejaculated sperm in summer versus winter seasons has been previously interrogated using the microarray technology (Yang et al., 2010). In that study the authors identified 33 dysregulated transcripts, none of which was differentially abundant in our dataset. This lack of concordance between works could be due to both biological and technical reasons and is somewhat expected. First, the two studies interrogated different animal populations in different geographic locations. The study by Yang et al. (2010) focused on Duroc boars breed in a sub-tropical region in Taiwan (25°N) whilst we screened Pietrain males from a sub-Mediterranean temperate climatic zone in Catalonia with warm summers and mildly cold winters (köppen classification Cfb; latitude 42°N). Moreover, we used a RNA-seq approach targeting the whole transcriptome whilst Yang et al. (2010) employed a custom microarray interrogating only 708 target genes and by large, ignored the vast catalog of annotated genes.

We also identified 5 miRNAs down- and 2 miRNAs up-regulated in winter (Table 4). This set included miR-34c, which was one of the most abundant miRNAs in our study, as well as in the sperm of other species, and was down-regulated in the winter samples. The RNA levels of miR-34c were also down-regulated in the sperm of men and mice exposed to severe early life stress events (Dickson et al., 2018), and in the testis of cynomolgus monkeys exposed to testicular hyperthermia (Sakurai et al., 2016), thus suggesting a link between the seasonality of semen quality and miR-34c. miR-1249, up-regulated in the winter group, was also found to be altered in the semen of bulls with moderate fertility (Fagerlind et al., 2015). Members of the miR-106 family were recently associated with oxidative stress in several tissues and cell types. For example, miR-106b targets the 12/15-Lipoxygenase enzymes, which are involved in the metabolism of fatty acids and oxidative stress in murine neurons (Wu et al., 2017). miR-106b has also been related to autophagy and cellular stress in intestinal epithelial HCT116 cells (Zhai et al., 2013). A study in cattle identified a single nucleotide polymorphisms in a miR-378 target site of the *INCENP* semen quality associated gene (Liu et al., 2016). In humans, miR-378 was found to also target the autophagy related protein 12 gene (*ATG12*) in cervical cancer (Tan et al., 2018). Finally, miR-221 was linked to autophagy in several tissues as well (Li et al., 2016; Qian et al., 2017) and was shown to regulate *SOD2*, which has key mitochondrial anti-oxidant functions in a murine model of ischemic skeletal muscle regeneration (Togliatto et al., 2013).

**Table 4:** List of the miRNAs showing distinct seasonal abundance.

miRNA ID	Log2 (FC)	*p*-value	*q*-value (FDR)
ssc-miR-221-3p	−2.70	4.19E-05	1.54E-03
ssc-miR-362	−1.81	1.63E-03	2.18E-02
ssc-miR-378	−1.71	6.16E-03	4.94E-02
ssc-miR-106a	−1.62	1.75E-05	1.29E-03
ssc-miR-34c	−1.53	5.87E-04	9.59E-03
ssc-miR-1306-5p	1.68	1.81E-04	3.81E-03
ssc-miR-1249	3.14	2.58E-08	3.79E-06

*The list includes only these miRNAs with q-value < 0.05 and log2FC > 1.5. FC, fold-change; FDR, false discovery rate.*

Our results are in consonance with previous reports suggesting that oxidative stress and autophagy are the key causes of the loss of semen quality in the warm summer periods (Suriyasomboon et al., 2006; Zasiadczyk et al., 2015). This data should be confirmed in a matched study where the winter and summer ejaculates come from the same boars using additional animals and several ejaculates per boar to account for non-genetic intra-individual variation.

## Conclusion

We have identified a rich and complex sperm transcriptome with known and novel coding RNAs, lncRNAs and sncRNAs that resembles the human, mouse and cattle counterparts. Their roles are mainly related to the regulation of spermatogenesis, fertility and early embryo development. These spermatozoal transcripts are fragmented, likely in a selective manner, consistently affecting some genes more than others across samples. This suggests that their fragmentation is not stochastic and follows an unknown deterministic pattern with potential functional implications. Similarly, the variability of the transcript abundance between samples was transcript specific. This in-depth transcriptome profile can be used as a reference to identify RNA markers for semen quality and male fertility in pigs and in other animal species.

Interestingly, the levels of some transcripts changed between the summer and the winter ejaculates, most likely responding to heat stress, which would in turn, cause oxidative stress, sperm membrane and DNA damage and autophagy. The biological basis of these transcriptome changes needs to be further explored. In the recent years it has become evident that the ejaculate contains different sub-populations of sperm, each with specific roles upon ejaculation. Each of these sub-populations may carry a specific transcriptome profile. Thus, the changes in transcript abundances that we identified may reflect either similar variations on the transcript’s profile in all spermatozoa cells or on the contrary, may be attributed to changes in the proportion of sperm sub-populations each carrying their specific transcript profile. Discriminating both hypotheses could help defining the best strategies to mitigate this seasonal effect. Single-cell RNA-seq, a novel and powerful technology that still needs to be optimized in spermatozoa, could allow identifying the sperm sub-populations and their relevance for seasonality, semen quality and fertility. In conclusion, our results pave the way to carrying future research to understand the molecular basis of semen quality seasonality in pigs, humans and other affected species.

## Ethics Statement

The ejaculates obtained from pigs were privately owned for non-research purposes. The owners provided consent for the use of these samples for research. Specialized professionals at the farm obtained all the ejaculates following standard routine monitoring procedures and relevant guidelines.

## Author Contributions

MG, AS, and AlC conceived and designed the experiments. SB collected the samples. JR-G carried the phenotypic analysis. MG performed sperm purifications and RNA extractions. AnC carried the qPCRs and their analysis. MG made the bioinformatics and statistic analysis. ME developed the SRE pipeline and provided bioinformatics support. MG analyzed the data, with special input from SK and AlC. MG and AlC wrote the manuscript. All authors discussed the data and read and approved the contents of the manuscript.

## Conflict of Interest Statement

The authors declare that the research was conducted in the absence of any commercial or financial relationships that could be construed as a potential conflict of interest.

## Footnotes

1 https://www.bioinformatics.babraham.ac.uk/projects/fastqc/

2 http://picard.sourceforge.net

## Supplementary Material

FILE S1 RNA-seq quality and mapping statistics. Average and Standard Deviation (SD) for the 10 boar sperm samples processed, including: amount of RNA extracted and several RNA-seq bioinformatics statistics for both total and small RNA-seq.

FILE S2 Distribution of the top decile most abundant SREs (Sperm RNA Elements) into SRE types and gene biotypes. Number of SREs (within the top decile) for each SRE type (exonic, intronic, upstream/downstream 10 kb and orphan). Total non-redundant number of genes and their biotype for each SRE class.

FILE S3 List of human and bovine genes identified by syntenic alignment of the orphan SREs. Orphan SRE genome coordinates were liftover to human and bovine coordinates, and the genes mapped in these regions were extracted. A total of 45 genes shared in both species were found. From these genes, 44 were already annotated in the *Sscrofa* Ensembl v.91 annotation. 17 of these genes were also detected by exonic, intronic and/or upstream/downstream 10 kb SREs. This suggests that orphan SREs could correspond to unannotated isoforms or to paralogous genes.

FILE S4 Gene Ontology analysis of the genes including the top decile most abundant and the orphan SREs detected in the SRE pipeline. GO biological process terms with significant Bonferroni corrected *p*-values (*p*-val < 0.05) and their associated genes.

FILE S5 Gene Ontology analysis of the different SRE abundance variance groups. GO biological process terms with significant Bonferroni corrected *p*-values (*p*-val < 0.05) and their associated genes.

FILE S6 Correlation between transcript integrity across samples, with transcript abundance and coding sequence length. Correlation of the TIN (Transcripts Integrity Number) between samples, with the transcript abundance and with the coding sequence length of the transcripts. This table shows the correlation of the TIN (Transcripts Integrity Number) between each pair of samples, the correlation of the TIN with the transcript average abundance in FPKM (Fragments per Kilobase per Million mapped reads) across the 10 samples, and the correlation of the TIN with the length of coding sequence of the transcripts.

FILE S7 Summary statistics of the *de novo* transcriptome assembly. Summary statistics of the Trinity output based on the number of potential novel genes and transcripts, and size (in bp) of the contigs based on all transcripts isoforms or based only on the longest isoform for each potential gene.

FILE S8 List of proteins identified by *de novo* analysis, with the species in which they were detected and transcript abundance. *De novo* analysis of the unmapped reads resulted in 1,060 proteins which passed the quality control filters. For each protein, we include the cognate species, the predicted RNA mean abundance in the 10 samples (in FPKM), the Standard Deviation (SD) of their RNA abundance and the gene ID symbol retrieved from Uniprot (https://www.uniprot.org/). FPKM: Fragments per Kilobase per Million mapped reads.

FILE S9 Non-redundant list of genes identified by *de novo* analysis. 768 potentially novel genes were identified from the unmapped reads. The gene symbol IDs were retrieved with Uniprot from the Trinity output protein names. These genes were detected in at least one species (detailed in column 2 of Supplementary File S8). The majority of these genes were annotated in the porcine Ensembl v.91 but 29 were identified as novel genes. 40 of the genes annotated in the porcine genome were not detected with the SREs pipeline which indicates that none of their cognate reads mapped to the genome even though these genes are annotated.

FILE S10 List of long non-coding RNAs detected in porcine sperm. Ensembl IDs of the lncRNAs identified in this study, their genome coordinates, average RNA abundance across the 10 samples and length. Most of the lncRNAs presented, as an average across all samples, low RNA abundances.

FILE S11 Distribution of the short RNA-seq reads mapping to different RNA types. Proportion and standard deviation (SD) across the 10 samples.

FILE S12 Concordance of miRNA identification between our dataset and other sperm RNA-seq studies. Comparison of the miRNAs identified in our study with other sperm RNA-seq experiments in pig, in human, and cattle.

FILE S13 RNA abundance levels and coefficient of variation of miRNAs, tRNAs, and piRNAs in the porcine sperm. RNA abundance is measured in CPM (Counts Per Million) across the 10 samples. We only considered the miRNAS with >0 CPMs in all the samples. The genomic coordinates of piRNAs refer to the Sscrofa10.2 built instead of Sscrofa11.1 as provided by the piRNAs cluster database [40].

FILE S14 Novel piRNA clusters identified in the pig sperm RNA. We detected 17 potential clusters of piRNAs that were found in at least 3 of the 10 samples analyzed in this study. Mean and standard deviation (SD) in CPM (Counts Per Million).

## Abbreviations

CPM: counts per million
CV: coefficient of variation
FPKM: fragment per kilobase per million mapped reads
LINE1: long interspersed nuclear element 1
lncRNAs: long non-coding RNAs
miRNAs: micro RNAs
NGS: next generation sequencing
piRNAs: piwi-interacting RNAs
RE: repeat element
RPKM: reads per kilobase per million mapped reads
SINE: short interspersed nuclear element
sncRNAs: small non-coding RNAs
SRE: sperm RNA element
TIN: transcript integrity number
tRNAs: transfer RNAs.
